# Stimulus Saliency Modulates Pre-Attentive Processing Speed in Human Visual Cortex

**DOI:** 10.1371/journal.pone.0016276

**Published:** 2011-01-21

**Authors:** Thomas Töllner, Michael Zehetleitner, Klaus Gramann, Hermann J. Müller

**Affiliations:** 1 Department of Psychology, Ludwig-Maximilians-University of Munich, Munich, Germany; 2 Swartz Center for Computational Neuroscience, Institute for Neural Computation, University of California San Diego, La Jolla, California, United States of America; 3 Department of Psychological Sciences, Birkbeck College, University of London, London, United Kingdom; Ecole Polytechnique Federale de Lausanne, Switzerland

## Abstract

The notion of a saliency-based processing architecture [1] underlying human vision is central to a number of current theories of visual selective attention [e.g., 2]. On this view, focal-attention is guided by an overall-saliency map of the scene, which integrates (sums) signals from pre-attentive sensory feature-contrast computations (e.g., for color, motion, etc.). By linking the *Posterior Contralateral Negativity* (PCN) component to reaction time (RT) performance, we tested one specific prediction of such *salience summation* models: expedited shifts of focal-attention to targets with low, as compared to high, target-distracter similarity. For two feature-dimensions (color and orientation), we observed decreasing RTs with increasing target saliency. Importantly, this pattern was systematically mirrored by the timing, as well as amplitude, of the PCN. This pattern demonstrates that visual saliency is a key determinant of the time it takes for focal-attention to be engaged onto the target item, even when it is just a feature singleton.

## Introduction

Every single second, the human vision system takes in approximately 10^7^ to 10^8^ bits of information [1] transmitted via the optic nerve. From this enormous data pool (and in addition to the data from the other senses), we need to select relevant or salient information in order to determine adequate actions and control their execution. Due to our inability to process all incoming information at once, we typically resolve this data overload by selectively attending to individual objects in the scene, deploying attention serially from one object to another (see, e.g., Wolfe's Guided Search, GS, theory [2]).

One prominent conception of how visual selection is accomplished refers to the notion of a *saliency map* [3]. In this framework, the external world is initially registered by a set of dimensionally organized feature analyzer units (e.g., for color, orientation, motion). Each dimensional module encodes the presence of feature contrast for all locations across the visual field, with feature contrast computations being modulated by the spatial separation between neighboring items [4]. The feature contrast signals are then integrated across dimensions, in a location-specific manner, by units in an *overall-saliency map* of activations; again, this integration, or *saliency summation*, process is spatially scaled [5]. Following this, the most active unit on this map is determined in a competitive, winner-take-all process, and focal attention will be deployed to the location represented by this unit. Focal-attentional selection, in turn, mediates high-level stimulus analysis and response decision processes. Importantly, an active overall-saliency map unit signals only that there is a feature difference at the location it represents relative to surrounding locations, but not what the identity (dimensional, featural) of the stimulus is that gives rise to this signal. Consequently, stimulus identification requires gradual backtracking, by recurrent processes, to hierarchically lower stages (dimensional feature contrast and feature maps) in order to ‘extract’ the information of interest [6]–[8]. Thus, on this ‘classical’ view, the saliency map represents the physical (bottom-up) distinctiveness of objects in the visual scene; signaling supra-dimensional feature contrast rather than absolute feature values at a given location relative to its surround [9]. However, these signals may be also top-down modulated, at least to some extent, based on observers' stimulus expectancies [1], [8], [10].

Over the last two decades, a large amount of research has been devoted to identifying the neural correlates of such a (putative) saliency map in *non-human primates* as well as *humans* based on single-cell recordings [11]–[18], functional magnetic resonance imaging (fMRI) [19], [20], and repetitive transcranial magnetic stimulation (rTMS) [21], [22]. For instance, Sato and colleagues [16], [23] have demonstrated that manipulating target-distracter similarity in a visual search task had a strong impact on the time required by monkey FEF neurons to dissociate a target from the distracters. Furthermore, Beck and Kastner [19] recorded hemodynamic brain responses for stimulus arrays in which a single item differed in color and orientation from its surrounding three items (i.e., homogeneous ‘pop-out’ displays), compared to when all four items differed in color and orientation from each other (heterogeneous displays). Beck and Kastner found that suppressive sensory interactions that usually arise from simultaneously presented multiple stimuli are eliminated at the level of V2/VP and V4 when one of the items was a feature singleton. Thus, extrastriate areas seem to play a significant role in biasing the selection of salient visual stimuli (for V1 modulations, see also [24]). Further neural populations that provide topographic representations of the external space have been identified, for instance, in the inferior and lateral subdivisions of the pulvinar [25], the superior colliculus [26], lateral intraparietal cortex [27], and in dorsal-stream areas [28]. In sum, a number of neurological results indicate that multiple areas along the visual processing pathways encode stimulus saliency, pointing to the possibility of a distributed saliency map [29]. While this is in general agreement with the notion of a saliency-based processing architecture, in which saliency signals are computed at different hierarchical levels, it remains an open issue whether these computations are integrated by an overall-saliency map, thereby modulating visual selection times.

**Figure 1:**
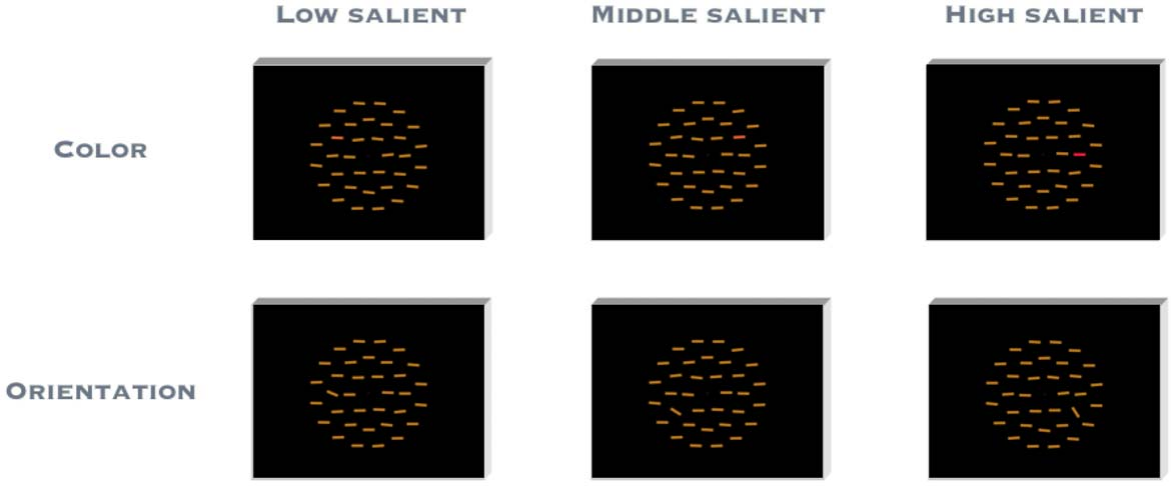
Stimulus displays used in the present visual pop-out binary localization task. Participants were required to give a speeded forced-choice response indicating the position (left vs. right hemi-field) of the feature singleton, which was selected randomly from one of the six lateral positions on the middle circle.

To investigate the temporal dynamics underlying the formation of salience representations in the *human* visual system, we recorded electro-cortical brain responses (the electroencephalogram, EEG) during a visual feature singleton (i.e., pop-out) search task. Specifically, we tested a direct implication of *salience summation* models, such as the dimension-weighting account (DWA) of Müller and colleagues [7], [30]–[32]. In contrast to, for instance, interactive race [33] or serial (exhaustive) processing architectures (e.g., Feature Integration Theory [34]), the DWA assumes that selective (focal) attention operates on a map of cross-dimensionally integrated (summed) feature contrast signals, with the most active location on this overall-saliency map determining the allocation of focal attention. Thus, presentation of high-, as compared to low-, salient pop-out targets should result in the computation of stronger feature contrast signals in early sensory coding, which in turn would speed up the accrual of activation at the level of the overall-saliency map. According to the DWA, this should result in *faster* focal-attentional target selection and, thus, expedited reaction times (RTs). Note that lower levels of feature contrast are often assumed to produce inefficient search, where the likelihood that the target is the first item selected is reduced, due to noise in feature contrast computation, and consequently RTs increase with increasing number of items in the display [18], [35]–[37]. Recently, however, Zehetleitner and colleagues [38], [39], [62] have shown that a reduction in feature contrast can lead to slower RTs without making the search inefficient. The contrast levels used in the present study lie within the range in which the probability that the target will be selected as the first item is constantly one, but where the time required for selection is modulated (see figure 1).

To assess whether the initiation of focal-attention shifts is indeed determined by visual (target) saliency in feature singleton searches, we specifically focused on the *Posterior-Contralateral Negativity* (i.e. PCN) component of the event-related potential (ERP), which has been demonstrated [41]–[43] to reflect visuo-spatial attention shifts based on perceptual stimulus attributes. Traditionally, this component has been referred to as *N2-posterior-contralateral* (N2pc); however, given its clear-cut independence, in terms of timing and activation, of the non-lateralized N2 [40], we prefer to use the term PCN (instead of N2pc) in order to prevent misleading associations. The PCN is typically observed as a negative-going deflection over visual brain areas of the hemisphere contralateral to the location of an attended stimulus approximately between 175 and 300 ms post stimulus. Combining event-related magnetic fields, fMRI, and ERPs, Hopf and colleagues [44] have recently shown that the neural generators underlying this component are sited within the human homologues of monkey inferotemporal cortex and area V4. Interestingly, most previous ERP studies focused solely on the activation and/or presence (versus absence) of this component in order to study covert attention shifts and/or the extent of attentional-resource allocation. In fact, most of these studies implicitly assumed that the timing of the PCN is constant and linked to the non-lateralized N2 component. Recently, however, a growing number of studies [31], [45], [46] has specifically concentrated on the timing of the PCN, which can be taken as a (temporal) marker of the transition from the pre-attentive sensory coding of the whole stimulus display to the focal-attentional selection and analysis of the selected item. Thus, it has been demonstrated that the *speed* of visual target selection varies dependent on, for instance, stimulus intensity [47], set size [48], the dimensional identity of the target on the previous trial [31], and the definition of a feature singleton in one versus multiple (redundant) feature dimensions [46].

Linking the theoretical implications of a salience summation architecture to event-related brain potentials, which permit a millisecond-by-millisecond measure of neural processing based on scalp-recorded voltage fluctuations, we hypothesized that the *timing* of the PCN component should vary systematically as a function of the visual saliency of feature singletons, thus reflecting their differential encoding rates in the pre-attentive, feed-forward sweep of visual processing. That is, the more a (pop-out) target differs from its surround, the *earlier* the PCN should be triggered – which, when cascaded forward to later, response-related processes, should yield *speeded* RT performance.

## Materials and Methods

### Participants

Thirteen observers (4 female) took part in this experiment. Their ages ranged from 20 to 30 (median 25) years. All had normal or corrected-to-normal vision and reported no history of neurological disorders. Observers gave their written informed consent and were either paid or received course credit for participating. The experimental procedure was approved by the ethics committee of the Department of Psychology, University of Munich, in accordance with the Code of Ethics of the World Medical Association (Declaration of Helsinki).

### Stimuli, Task, and Study Design

The visual search display consisted of 34 colored bars (0.6° of visual angle high×2.7° wide) presented against a black background. The stimuli were arranged around the circumferences of three imaginary (concentric) circles centered on a white fixation spot. The circles were 4.5°, 8.5°, and 12.5° of visual angle in radius and were made up of 6, 12, and 16 items (inner, middle, and outer circle), respectively. On each trial, the stimulus display contained a singleton target, amongst 33 distracters, that was equally often defined in the color and, respectively, the orientation dimension. All distracter bars were yellow (CIE .456, .469, 23) and oriented horizontally (i.e. 90° tilt to the vertical) and independently uniformly randomly jittered in tilt ±7° (to the horizontal, see Figure 1). Target-distracter similarity was parametrically manipulated using three different feature-contrast levels for color (high: CIE .595, .332, 23; intermediate: CIE .555, .367, 23; low: CIE .540, .388, 23) and orientation targets (high: 33.5°; intermediate: 58°, low: 67° tilt to the vertical), all with equal probability. A control experiment verified that the targets of low as well as those of intermediate and high feature contrast in the orientation and color dimensions were indeed found efficiently. In this experiment, target-absent trials were mixed with trials in which displays contained either an orientation or a color target of either the highest or the lowest feature contrast used in the main experiment. Observers indicated target presence by a speeded mouse click (withholding a response on target-absent trials). To estimate search efficiency, set size (i.e., the number of items in the display) was either 7 or 19 items. The slopes of the functions relating search RTs to set size were shallow with both high- and low-feature-contrast targets (range between −0.2 and 1.5 ms/item). All slopes were significantly less than 5 ms/item (all p<.003) – a generally agreed criterion for efficient search [49].

The position of the target singleton was selected randomly from one of the six lateral positions on the middle circle. Observers were instructed to maintain central fixation throughout the experiment and to give a speeded forced-choice response indicating the location (left vs. right) of the singleton, by pressing the corresponding mouse button (left- vs. right-hand thumb response). Note that on notions deriving from Feature Integration Theory [34], [50], detection of feature-based signals may be mediated by a route that is separate from a localization route. However, recent work [51] has shown that even detection responses exhibit spatial characteristics. In addition, Töllner and colleagues [52] found that the (timing and amplitude of the) PCN is elicited absolutely independent of whether the target had to be detected or localized. This indicates that the same spatial mechanism or representation (e.g., saliency map) underlies both detection and localization responses (thereby refusing the notion of dual routes).

The experiment was conducted in a dimly illuminated, sound-attenuated, and electrically shielded cabin (IAC). The stimuli were presented on a 17″ computer screen, placed at a distance of approximately 75 cm to the observer. One experimental session consisted of 24 blocks of 72 trials each, resulting in a total of 1728 trials. A trial started with the presentation of the central fixation point for 500 ms, followed by the search display for 200 ms. Trials were terminated by the observer's response or after a maximum duration of 1000 ms. During the inter-trial interval (ITI), the fixation point was visible for a variable duration between 950 and 1050 ms (uniformly distributed). In case of a response latency longer than 1000 ms or a wrong response, the word ‘FEHLER’ (German word for ‘error’) was centrally presented for 1000 ms, providing direct response speed and error feedback. Prior to the start of the experiment, one block of practice was performed to familiarize observers with the stimuli and the stimulus-response mapping. After each block, participants received summary performance statistics (mean error rate and reaction time) as feedback information.

### EEG Recording and Data Analysis

The electroencephalogram (EEG) was recorded continuously from 64 scalp sites at a digitization rate of 1000 Hz. Electrodes were mounted on an elastic cap (Easy Cap, FMS), with positions corresponding to the 10-10 System [53]. Horizontal and vertical EOG was monitored by means of electrodes placed at the outer canthi of the eyes and, respectively, the superior and inferior orbits. All electrodes were referenced to Cz and re-referenced offline to linked mastoids. Impedances were kept below 5 kΩ. Electrophysiological signals were amplified using a 0.1–250-Hz bandpass filter using BrainAmp amplifiers (BrainProducts, Munich) and filtered offline with a 1–40-Hz band-pass (Butterworth zero phase, 24 dB/Oct). Prior to epoching the EEGs, an independent-component analysis (ICA), implemented in the Brain Vision Analyzer software (BrainProducts, Munich), was run to identify and backtransform components that represent blinks and/or horizontal eye movements. The EEG was then epoched into 500-ms segments relative to a 200-ms baseline, which was used for baseline correction. Only trials with correct responses and without artifacts – defined as any signal exceeding ±60 µV, bursts of electromyographic activity (permitted maximal voltage steps/sampling point of 50 µV), and activity lower than 0.5 µV within intervals of 500 ms (indicating dead channels) – were selected on an individual-channel basis, prior to averaging. The PCN component was quantified by subtracting ERPs obtained at lateral posterior electrode positions PO7/PO8 ipsilateral to the side of the singleton in the search array from contralateral ERPs. PCN latencies were determined individually as the maximum negative deflection in the 150–350-ms time window post stimulus. PCN amplitudes were calculated averaging five sample points before and after the maximum deflection. PCN onset latencies were estimated based on Ulrich and Miller's (2001) jackknife-based scoring method, which defines the onset as the point in time at which the amplitudes reaches a specific criterion relative to the pre-stimulus baseline. As suggested by Ulrich and Miller [54], we used 50% maximum amplitude as an optimal criterion for determining the onset of stimulus-locked ERP potentials. Electrophysiological (latencies, onset latencies, and amplitudes of the PCN) as well as behavioral measures (reaction times, error rates) were subjected to two-way repeated-measure analyses of variance (ANOVAs) with the factors Dimension (color, orientation) and Saliency (high, middle, low). Significant main effects and/or interactions were further examined using Tukey HSD post-hoc comparisons.

## Results

### Behavioural Data

**Figure 2:**
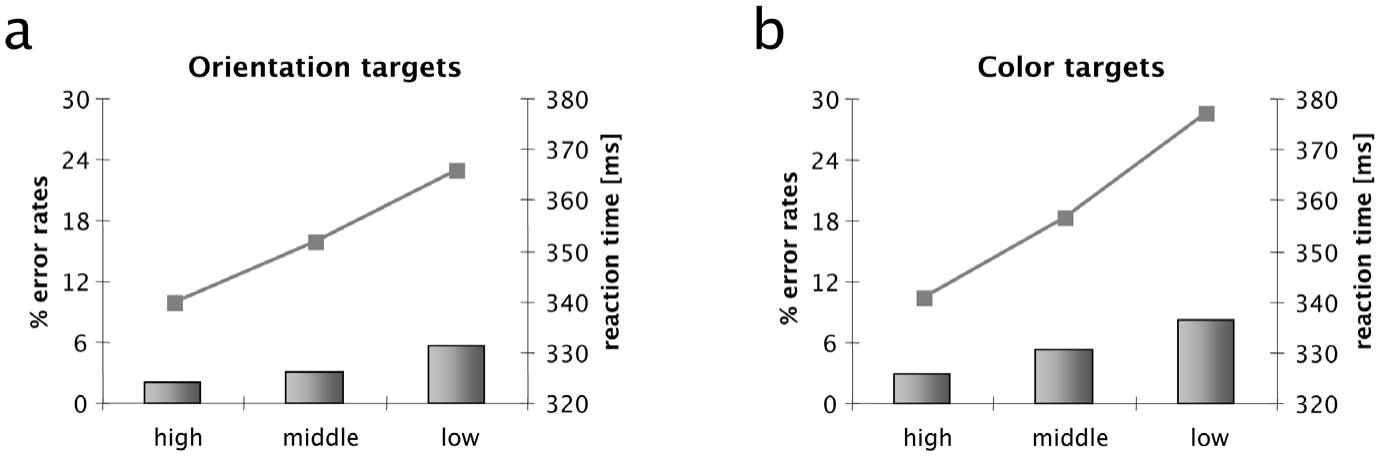
Behavioural results. (a) Reaction times (lines) and error rates (bars) as a function of Saliency (High, Middle, Low) for orientation-defined (pop-out) targets. (b) Reaction times (lines) and error rates (bars) as a function of Saliency (High, Middle, Low) for color-defined (pop-out) targets.

Figure 2 presents the error rates and reaction times separately for orientation (Figure 2a) and color targets (Figure 2b). For both feature dimensions, participants made the fewest errors when the target was highly salient (2,5%), with a gradual increase for targets of intermediate (5.4%) and low saliency (7.0%): F(2,22) = 31.5, p<.0001. Furthermore, error rates were overall higher for color than for orientation targets (5.6% vs. 3.6%), F(1,11) = 27.3, p<.0003, with the effect of the feature contrast manipulation being more pronounced for the color than for orientation: F(2,22) = 3.8, p<.04. Subsequent post-hoc contrasts (Tukey HsD) confirmed error rates for all feature contrast levels to differ significantly from each other.

A similar pattern was obtained for the reaction times (RTs). As illustrated in Figure 2, RTs were fastest for high-saliency targets (340 ms) and increasing gradually for targets of intermediate (354 ms) and low saliency (372 ms): F(2,22) = 104, p<.0001. Furthermore, RTs were overall faster for orientation (353 ms) than for color (359 ms): F(1,11) = 7.4, p<.02, and the effect of saliency was more pronounced for color than for orientation targets (two-way interaction): F(2,22) = 7.4, p<.004. Post-hoc contrasts revealed all feature contrast levels to differ significantly different from each other.

### Electroencephalographic Data

**Figure 3:**
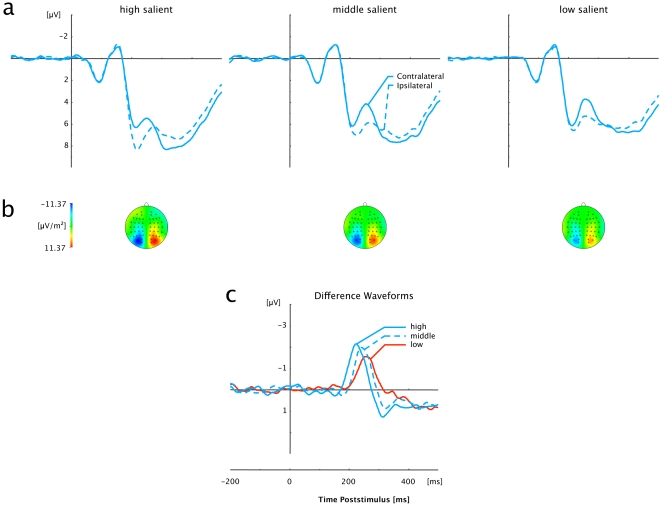
Grand averaged event-related brain potentials elicited in response to color-defined (pop-out) targets at electrodes PO7/PO8. (a) Waveforms contra- and ipsilateral to the singleton location. (b) Topographical maps of PCN scalp distributions for each of the three Salience conditions (High, Middle, Low) at the point in time when the difference between contra- and ipsilateral waveforms reached its maximum. These maps were computed by mirroring the contra-ipsilateral difference waves to obtain symmetrical voltage values for both hemispheres (using spherical spline interpolation). (c) PCN difference waves obtained by subtracting ipsilateral from contralateral activity for each of the three Salience conditions (High, Middle, Low).

**Figure 4:**
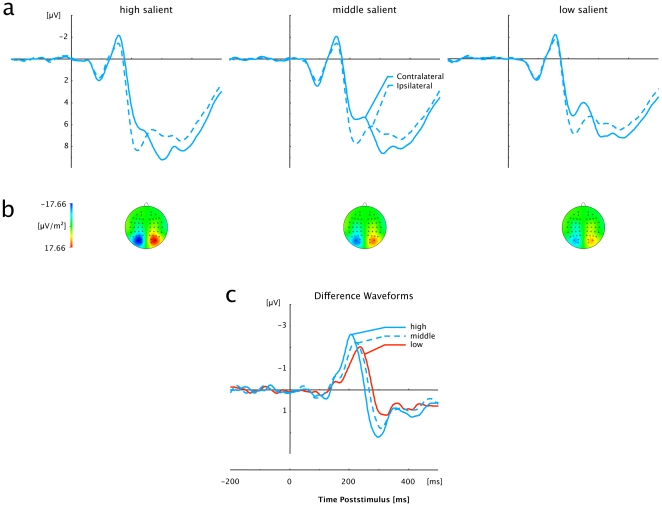
Grand averaged event-related brain potentials elicited in response to orientation-defined (pop-out) targets at electrodes PO7/PO8. (a) Waveforms contra- and ipsilateral to the singleton location. (b) Topographical maps of PCN scalp distributions for each of the three Salience conditions (High, Middle, Low) at the point in time when the difference between contra- and ipsilateral waveforms reached its maximum. These maps were computed by mirroring the contra-ipsilateral difference waves to obtain symmetrical voltage values for both hemispheres (using spherical spline interpolation). (c) PCN difference waves obtained by subtracting ipsilateral from contralateral activity for each of the three Salience conditions (High, Middle, Low).

Grand average ERP waveforms elicited by visual displays that contained singleton (pop-out) targets of high, intermediate, and low saliency are shown separately for color and orientation singletons in Figure 3 and 4, respectively. Separate waveforms for contra- and ipsilateral targets with respect to the hemisphere of the recording electrode (PO7/PO8) are shown in the top panels, while the bottom panels present the corresponding contralateral-minus-ipsilateral difference waveforms. For all six (dimension×saliency) experimental conditions, a solid PCN was triggered, which is evident as a more negative (i.e., less positive) voltage starting within a time window approximately 150 to 200 ms post stimulus.

As can be seen from these figures (bottom panels), for both feature dimensions, the rise of the PCN occurred the earlier and was the more pronounced the more the target differed from its distracter surround. These observations are substantiated by significant main effects of Saliency for PCN onset latencies, PCN peak latencies, and PCN amplitudes [*onset latencies:* F_c_ (2,22) = 47.799, p_c_<.001; *peak latencies:* F(2,22) = 70.563, p<.001, η^2^ = .865; see below for the amplitude effect]. Statistically, the shortest onset and peak latencies were observed for high-saliency targets (onset: 187 ms; peak: 223 ms), followed by targets of intermediate (203 ms; 240 ms) and of low saliency (215 ms; 250 ms); post-hoc comparisons confirmed all (saliency) conditions to differ significantly different from each other (p<.001). In addition, peak and onset latencies differed with respect to the dimensional identity of the target [*onset latencies:* F_c_(1,11) = 48.310, p_c_<.001; *peak latencies:* F(1,11) = 17.894, p<.001, η^2^ = .619], with shorter latencies for orientation (190 ms; 229 ms) than for color targets (214 ms; 247 ms). Note that the interactions between the two factors were non- significant [*onset latencies:* F_c_ (2,22) = 1.223, p_c_>.314; *peak latencies:* F(2,22) = 0.105, p>.901, η^2^ = .009], suggesting that the saliency-dependent timing of the PCN can be generalized across (at least) these two visual dimensions.

Furthermore, the degree of target-distracter similarity was signalled by the PCN amplitudes [F(2,22) = 7.135, p<.004, η^2^ = .393], with the strongest activations for high-saliency targets (−2.64 µV), and gradually decreasing activations for intermediate- (−2.37 µV) and low-saliency targets (−2.08 µV). Post-hoc analyses revealed only the difference between high- and low-saliency targets to be significant (p<.002; p>.148 for comparisons involving intermediate-saliency targets). In contrast to the PCN latencies, there was no influence of the target-defining dimension on the PCN activations, as evidenced by the absence of a significant effect involving Dimension [main effect: F(1,11) = 1.032, p<.332, η^2^ = .086; Saliency×Dimension interaction: F(2,22) = 1.024, p>.376, η^2^ = .085].

## Discussion

When introducing the idea of a *saliency map* in 1985, Koch and Ullman inspired researchers across disciplinary boundaries in cognitive, neurophysiological, and computational sciences. Two and a half decades later, a large body of studies [11]–[28] has revealed the neural expression(s) of saliency by means of single-cell recordings, fMRI, and TMS in the non-human primate as well as the human visual system.

Here, we have reported *electro-cortical* evidence for modulations of pre-attentive processing speed as a function of visual target saliency (high<middle<low) in the *human* visual cortex. For two distinct feature-dimensions (color and orientation), we found visual saliency to systematically influence the timing (and amplitude) of the *Posterior Contralateral Negativity* – a component generally agreed to reflect focal-attention shifts in visual space [41]–[43]: the shortest (onset and peak) latencies were elicited in response to high-saliency feature singletons, with a graded increase in PCN latencies for singletons of intermediate and low saliency. Importantly, this electrophysiological activation pattern was systematically mirrored by the latencies of the behavioral RTs, demonstrating that the salience of a given stimulus plays a crucial role for the time it takes for focal attention to be engaged onto the target object, even when the target is just a feature singleton.

Our results are closely in line with Guided Search-type of models (e.g., GS [2]; dimension-weighting account; [30]), according to which this saliency-based activation pattern arises at the stage of pre-attentive visual coding. That is, early sensory feature detection mechanisms (e.g., for color, orientation, motion) are assumed to compute feature contrast (i.e., dimensional saliency) signals for all locations across the visual field. These signals are then passed to a common master map of locations; with each unit accumulating activity towards a *threshold* [55], [56]. The unit that reaches the threshold first triggers a shift of focal-attentional processing resources to the location it represents, thereby mediating deeper and explicit stimulus analyses (e.g., feature identification) and motor response decisions. Accordingly, high- (relative to intermediate- and low-) saliency targets give rise to enhanced coding of feature contrast signals at pre-selective processing stages, which – cascaded forward to later response-related processes – leads to speeded RTs.

This saliency-based activation pattern of the PCN complements a series of recent EEG studies that specifically focused on the timing of this component in order to dissociate pre-attentive versus post-selective contributions to (extensively studied) RT effects. Based on PCN latency variations, these studies revealed pre-attentive perceptual encoding stages to be involved in the generation of redundancy gains by pop-out targets defined in multiple dimensions [46], dimension-specific intertrial (dimension repetition vs. change) effects [31], as well as (semantic) dimensional pre-cueing effects [32]. Crucially, all these effects were likewise interpreted as arising from modulated saliency signals computed at the level of pre-attentive vision, indicating that the timing of the PCN reflects the speed of those coding stages that occur prior to, and provide the basis for, focal-attentional target selection.

In addition to the saliency-based PCN latency effects, the present data also revealed an amplitude effect: the greater the difference of the target from its surround, the larger the PCN amplitude. This effect of target-distracter similarity appears to be at variance with findings reported by Hopf and co-workers [57]. In their study, the stimulus displays comprised of twelve circles, each with differently colored upper and lower halves; there were two target circles, one in the left and one in the right (lower) visual field, both surrounded by five distracter circles. The task (Experiment 1) was to determine which of the two central target items contained a predefined color (e.g., yellow), with the response being based on the relative location of this color (upper vs. lower half) within the target item. Contrasting conditions in which target and distracters shared two features (color and orientation) as compared to only one feature (color), Hopf et al. observed enhanced neural activity underlying the PCN with greater feature overlap between the target and distracter items. They took this as evidence that the processes underlying the PCN reflect the suppression of distracter interference, so as to resolve ambiguous target feature coding [58]. The present study, however, used simple pop-out search displays in which the target was easily detectable based on high physical distinctiveness, without a need for deeper focal-attentional stimulus analysis. This contrasts with the displays used by Hopf et al., in which the target – defined by an instructed color, which was however shared by the surrounding distracters – could appear at one of two possible locations. To solve the task, participants had to first select and then attentionally analyze the target item (to determine the elevation, top vs. bottom, of the instructed color) before they could decide upon the appropriate motor response. Given this, the conclusion of Hopf et al. that enhanced PCN activations reflect suppressive processes engaged to attenuate distracter interference may be limited to the particular demands of their task. By contrast, searching visual scenes that contain a (relatively) salient feature singleton target reverses the PCN activation pattern, giving rise to stronger activations with reduced, rather than increased, target-distracter similarity.

Although the present findings indicate a major role of bottom-up processes in the elicitation of the PCN (for feature singleton search) on the basis of physical distinctiveness, it should be noted that the activation and timing of this difference waveform can be modulated by top-down factors, such as ‘task (feature) set’ [59] or ‘dimensional set’ [32], [60]. In a recent study by Eimer and Kiss [59], a PCN and, thus, visuo-spatial shifts of attention were observable only when the feature singleton was relevant to the current task. More specifically, participants were asked to discriminate the orientation of a target bar, which was preceded by a cue array in which the location of a feature singleton, such as a color-defined singleton, was unrelated to the position of the upcoming target item. Eimer and Kiss found that a color singleton cue triggered a PCN (associated with a behavioral spatial-cueing effect) only when the subsequent target was also a color singleton (among color-homogeneous nontargets), but not when it was the only (luminance-defined) onset item in the target display. This pattern suggests that attention shifts elicited by spatially uninformative feature singleton cues are contingent upon the stimulus parameters specified in the top-down task set [cf. 61]. Consistent with this observation, behavioral detection responses to a feature singleton have been found to be expedited – associated with shorter PCN latencies – when observers are provided in advance with a cue word (e.g., shape) that indicates the probable target-defining dimension on a given trial [32]. This further underscores the notion that top-down control processes are able to modify the time course of focal-attentional target selection by altering the initial feedforward sweep of visual processing.

In conclusion, the present findings advance our knowledge of how visual saliency affects the processing of feature singleton targets. Recording electroencephalographic brain responses, we found that the conspicuity of visual (pop-out) target objects was strongly tied to the timing and magnitude of the PCN component, indicating that focal-attention shifts were triggered as a function of visual (target) saliency. Given this, our findings provide further support for a saliency-based processing architecture of the human visual system, in which the outcome of early sensory feature contrast computations guides the deployment of focal-attentional processing resources.
